# Processing of Polysulfone to Free Flowing Powder by Mechanical Milling and Spray Drying Techniques for Use in Selective Laser Sintering

**DOI:** 10.3390/polym8040150

**Published:** 2016-04-19

**Authors:** Nicolas Mys, Ruben Van De Sande, An Verberckmoes, Ludwig Cardon

**Affiliations:** 1 Center for Polymer and Material Technologies (CPMT), Faculty of Engineering and Architecture, Ghent University, Technologiepark building 915, Zwijnaarde 9052, Belgium; ludwig.cardon@ugent.be; 2 INdustrial Catalysis and Adsorption Technology (INCAT), Faculty of Engineering and Architecture, Ghent University, Valentin Vaerwyckweg 1, 9000 Ghent, Belgium; ruben.vandesande@ugent.be (R.V.D.S.); an.verberckmoes@ugent.be (A.V.)

**Keywords:** polysulfone, microsphere, rotor milling, spray drying, ball milling, polymer characterization

## Abstract

Polysulfone (PSU) has been processed into powder form by ball milling, rotor milling, and spray drying technique in an attempt to produce new materials for Selective Laser Sintering purposes. Both rotor milling and spray drying were adept to make spherical particles that can be used for this aim. Processing PSU pellets by rotor milling in a three-step process resulted in particles of 51.8 μm mean diameter, whereas spray drying could only manage a mean diameter of 26.1 μm. The resulting powders were characterized using Differential Scanning Calorimetry (DSC), Gel Permeation Chromatography (GPC) and X-ray Diffraction measurements (XRD). DSC measurements revealed an influence of all processing techniques on the thermal behavior of the material. Glass transitions remained unaffected by spray drying and rotor milling, yet a clear shift was observed for ball milling, along with a large endothermic peak in the high temperature region. This was ascribed to the imparting of an orientation into the polymer chains due to the processing method and was confirmed by XRD measurements. Of all processed powder samples, the ball milled sample was unable to dissolve for GPC measurements, suggesting degradation by chain scission and subsequent crosslinking. Spray drying and rotor milling did not cause significant degradation.

## 1. Introduction

Selective laser sintering (SLS) is a process which builds 3D objects by selectively sintering successive layers of powdered material with a laser. Formerly used as a rapid prototyping method, in the last decades the technique gained interest as additive manufacturing (AM) method to build actual end-use parts [1,2]. The principal advantage of this printing form is the ability to create parts with greater complexity than conventional processes. No supports are needed as the powder bed itself acts as supporting structure that can easily be removed. Tooling costs are greatly lowered and parts can easily be modified or even switched during build-up. With the growing interest in SLS as printing process, the demand for materials with more diverse chemical and mechanical properties compared to the conventional powders has surfaced. Presently, polyamide (PA) powders make up the majority of the current market of polymeric materials for AM [3]. Adding new materials to the supply of processable polymers could substantially broaden the application window by widening the range of mechanical and thermal properties. This diversification of processable polymers is part of the solution to the rising demand. The availability of the material in powder form of proper particle size and suitable morphology forms another obstacle in acquiring new build material. For SLS, the ideal particle size falls within the range of 45–90 μm and has a spherical morphology [4,5]. Deviations from these guiding rules could result in bad packing density, greater porosity and as a result, a decline of the mechanical properties of the 3D object [6]. Polymeric materials, however, are for the most part only available in pellet form. In addition to that, there seems to be no processing method available that can achieve all required objectives.

Conventional ball milling—either cryogenically or at ambient temperature—often leads to particles with a too-wide size distribution and undesired morphology [7,8]. Furthermore, these methods often take a lot of time and tend to leave a large amount of waste material that was not fully fractionated. Despite this fact, this technique is commonly applied in the industry, albeit mostly at cryogenic temperatures to speed up the pulverization process. Several studies have already been performed on the influence of ball milling on the mechanical properties of the polymer in question [9], the intrinsic properties such as molecular weight [10] and thermal properties of the polymer [11], and its structural changes regarding amorphization [12] by mechanical treatment. Yet the examined polymers always portrayed semi-crystalline polymers. Literature addressing mechanical treatment of amorphous polymers remains scarce. For this reason, and as a basis of comparison, ball milling as a processing technique is included in this work. The use of spheronizers is a widely-known method for enhancing the morphology of the pellets, but it generally results in particles well beyond the desired range [13]. Rotor milling is a new mechanical processing technique that can prove to be a valuable alternative to the conventional techniques. This semi-continuous technique allows shorter residence time of the material, which shortens processing time and minimizes the possibility of material degradation. Furthermore, materials can be milled to a predefined range of particle sizes by using inset sieves. To that end, rotor milling is included in this study on polymer powderization methods and it is compared to ball milling as a benchmarking technique.

Other processing methods concern more physicochemical methods, like thermal induced phase separation (TIPS) [14], diffusion induced phase separation (DIPS) [15], evaporation phase separation (EPS) [16], and spray drying (SD) [17,18]. The latter can be seen as a special form of EPS. These methods are said to achieve more spherical particles, yet require vast amounts of solvents (and in some cases non-solvents) to induce the phase separation. From these techniques, spray drying seems to be a better approach to process the polymer pellets into a spherical free-flowing powder with the required dimensions, as it allows for a higher weight percentage to be processed and the evaporating solvent can easily be recovered using an inert loop [19]. Moreover, particle size and morphology are better controlled than by conventional milling techniques and size distribution can be made considerably narrower. For this reason, this paper focuses on SD as one of the physicochemical processing techniques.

Polysulfone (PSU) is selected as testing material since this amorphous polymer exhibits significantly different thermal and mechanical properties than PA and therefore could potentially broaden the application window of SLS. It is a rigid, high-strength and transparent polymer with very high dimensional stability and good chemical resistance over a broad pH-range [20]. It has a higher tensile strength (72 MPa) and Young’s modulus (2.5 GPa) than conventionally used PA materials and exhibits higher thermal properties (Heat Deflection Temperature (HDT) of 175 °C and degradation temperature of 475 °C) [20]. This makes the polymer ideal for many applications involving the transport of liquids like faucets in the case of hot water, piping systems in the case of chlorinated water under pressure, or battery cases containing corrosive products. Additionally, their ability to be sulfonated (to PSU–SO_3_H) makes them ideal for ion exchange membranes [21,22].

In this article, PSU is processed from pellet to powder form via a physicochemical route (spray drying) and two mechanical routes (ball milling and rotor milling). The formed particles are compared in size and shape and investigated for the effect each processing technique imparts on the material itself. A degradation study using GPC measurements and viscosimetry as well as thermal analysis by DSC will be discussed in this regard.

## 2. Materials and Methods

### 2.1. Materials

Polysulfone Udel P-1700 was provided by Solvay (Brussels, Belgium) in the form of pellets and was used as-received. The pellets were dissolved in *N*,*N*-dimethylformamide (DMF, purity 99+ %, VWR, Leuven, Belgium).

### 2.2 Solubility Determination

A parameter based approach was selected using the Hansen solubility Parameter (HSP) model in order to find the most suitable solvent to dissolve PSU. The solubility of the given PSU polymer was determined gravimetrically as the minimum solvent weight necessary to completely dissolve a weighted sample of PSU. The solubility was expressed in weight percentage (wt %). Polysulfone was refluxed at 153 °C (boiling point of DMF) until dissolved and left to cool at room temperature. To minimize the experimental errors, the gravimetric experiment was replicated three times.

### 2.3. Viscosity Determination

Viscosity measurements were performed on a Thermo Scientific HAAKE viscotester 550 rotational viscometer (Thermo Scientific, Breda, Netherlands) according to ISO 3219. Samples were conditioned at constant temperature of 25 °C by a HAAKE K15 thermostatic bath (Thermo Scientific, Breda, Netherlands) and measured at 10 rounds per min using the MVI spindle. Each sample was measured at least three times. The results for the viscosity were then averaged.

### 2.4. Spray Drying

Spray drying was performed on a Buchi B290 (BÜCHI Labortechnik GmbH, Hendrik-Ido-Ambacht, Netherlands) equipped with a two-fluid nebulizer connected to pressurized air. The nozzle orifice size was 2.0 mm. The aspirator ran at a maximum air velocity of 40 m³/h. The heater inlet temperature, temperature of solution, solution feeding rate and gas flow rate were fixed factors in this article. The optimization of the morphology of the particles encompasses the variation of these parameters in a systematic approach and lies beyond the scope of this article. Details of the optimization process of the spray drying parameters will hence be reported elsewhere. For this purpose a fixed set of parameters is used based on previous obtained results [23].

A PSU solution of 12 wt % in DMF was heated to 150 °C and fed to the nozzle at a feed rate of 7.4 mL/min. The prepared solution was heated to decrease the solution viscosity and hence increase the diffusion rate of the solutes during drying. The heated solution was then atomized by the two-fluid nozzle into the drying chamber where the droplets were dried by the use of dry air at 210 °C.

The optimization of the morphology of the particles encompasses the variation of these parameters in a systematic approach and lies beyond the scope of this article. Instead we will focus on this set of parameters which has proven to give good jetting behavior with abundant spherical particles. Further optimization will be discussed in future work.

### 2.5. Mechanical Milling

#### 2.5.1. Ball Milling

Ball milling was performed on a Fritsch Pulverisette 500 planetary ball mill (Fritsch, Idar-Oberstein, Germany) with three ceramic cups and balls of 25 mm. The cups were loaded with 10 ± 0.02 g of PSU pellets. Communition took place at 1400 RPM and samples were taken at constant intervals (15 min) to be examined for size and morphology. The minimum communition time to achieve particles with the desired size was estimated in this manner. Heat build-up during communition was minimized by letting the powders condition to room temperature after each sampling run.

#### 2.5.2. Rotor Milling

Rotor milling was performed on a Fritsch Pulverisette 14 rotor mill (Fritsch, Idar-Oberstein, Germany) to pulverize the polysulfone pellets by a three-step communition process. In the first step the pellets were pulverized to a coarse powder using a sieve with mesh size 500 μm. In a second and third refinement step the coarse powder was further pulverized to a fine powder and sieved at 120 and 80 μm, subsequently. A 12-ribbed rotor blade was used at 15,000 RPM to achieve pulverisation. The resulting powder was sieved and isolated using a cyclone system. During the milling process the rotor mill was cooled by air at room temperature using an aspirator connected to the cyclone system.

### 2.6. GPC Measurements

GPC was used as a direct way to measure degradation by calculating the molecular weight of the virgin and processed samples. GPC measurements were performed on a Waters Instrument, with RI detector (2414 Waters), equipped with three Polymer Standards Services GPC serial columns (1 × GRAM Analytical 30 Å, 10 μm, and 2 × GRAM Analytical 1000 Å, 10 μm). PMMA standards (690 to 1,944,000 g·mol^−1^) were used for calibration and DMA containing LiBr (0.42 g·mL^−1^) was used as a solvent at a flow rate of 1 mL·min^−1^. Molecular weight and dispersities were determined using Empower software. Samples were injected with a PL-AS RT autosampler. Infrared measurements were carried out with an ATR Perkin Elmer FT-IR spectrum 1000 (Perkin Elmer, Zaventem, Belgium) in a range of 4000 to 600 cm^−1^. Each sample was measured twice.

### 2.7. DSC Measurements

The thermal properties of the produced powders were analysed using a Netzsch DSC 204F1 (NETZSCH-Gerätebau GmbH, Wolverhampton, UK) under nitrogen atmosphere. Samples were contained in an open Aluminum pan and referenced against an empty open Aluminum pan. A heating rate of 10 °C/min was used to heat the DSC to 450 °C to determine the effects of degradation and thermal history imparted during processing. A second heating run under the same conditions was performed to determine if any change in thermal properties had occurred. A baseline subtraction was done to correct for any slope or variation in heat transfer effects by performing the same measurement with an empty pan both in the reference and sample position and then subtracting the resultant curve. The sample material never exceeded 1.2% of initial sample weight (25.8 mg).

### 2.8. Particle Size Distribution (PSD)

The morphology of the produced particles was investigated using a scanning electron microscopy (JEOL JSM-7600F, JEOL Europe bv, Zaventem, Belgium) at low voltage (2 kV) and working distance of 8 mm. Samples were sputtered shortly with gold using a BAL-TEC SCD 005 Sputter Coater (Bal-tec GmbH, Wetter, Germany) at 25 mA. In some cases optical microscopy (Keyence digital microscope VHX-500F, Keyence International, Mechelen, Belgium) was used to determine size and shape of the particles. Obtained micrographs were then analysed using the software program Image J and further investigated using the statistical program SPSS.

### 2.9. XRD Measurements

Possible oriented structures were analysed by XRD analysis. XRD characterization was executed using a Thermo Scientific ARL X′tra X-ray diffractometer (Thermo Scientific, Breda, Netherlands) with the CuKα line as the primary source. The obtained diffractograms were compared to the diffractogram of unprocessed PSU. This was done to find out if any peaks arose in the spectrum as these are indicative of some ordered state.

## 3. Results

### 3.1 Solubility Determination

The HSP model is an extension of the Hildebrand solubility parameter model and gives an indication of the solubility of a material in a certain solvent. It is correlated to the cohesive energy density; the energy necessary to completely remove all intermolecular forces in a unit volume for a material to dissolve [24]. The total HSP (δt) comprises the contribution of a dispersive term (δd), a polar term (δp), and a term related to the effect of hydrogen bridges on the cohesive energy (δh). Hansen states that when the difference between solubility parameters of solvent and polymer is small (typically <4 MPa^1/2^) miscibility occurs and dissolution takes place. This theory has already been discussed in a previous paper [25] and therefore will not be elaborated upon in this article. Based on the Hansen Solubility Parameter (HSP) model, DMF was chosen as a suitable solvent for PSU.

One should note, however, that although the HSP model is in closer agreement with the experimental data, it still cannot fully describe the solution thermodynamics for every system—experimental validation is advised. Using this parameter, several solvents were screened, a section of which is displayed in Table 1. From this list, DMF was experimentally determined to be the most suitable solvent. Gravimetric experiments revealed that a maximum weight percentage of 18.18 wt % PSU or a solubility of 0.21 g/mL was attainable. The experiments were repeated thrice and each time the same result was obtained.

### 3.2. Viscosity Determination

**Figure 1:**
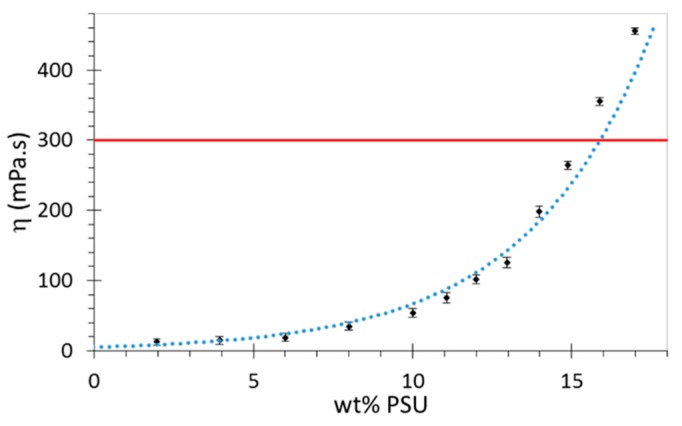
Viscosity curve of PSU solutions in *N*,*N*-Dimethylformamide (DMF) as a function of mass percent of PSU measured at 25 °C. The two-fluid nozzle theoretically only allows the spraying of solutions lower than 15 wt % (solid line).

Viscosity measurements were performed on a rotational viscometer to determine the maximum concentration that can be sprayed using the two-fluid nozzle system. Theoretically, viscosities of up to 300 mPa.s (solid line in Figure 1) are eligible for spraying using the two-fluid nozzle [26]. Any solutions displaying higher values would result in viscous dissipation at the nozzle head and simply drip off as a result of mass buildup. In reality, no solutions over 14 wt % were sprayed due to the formation of threadlike structures in the drying chamber. The properties of the solutions are displayed in Figure 1. In this article, only 12 wt % solutions were sprayed.

### 3.3. Morphology

**Figure 2:**
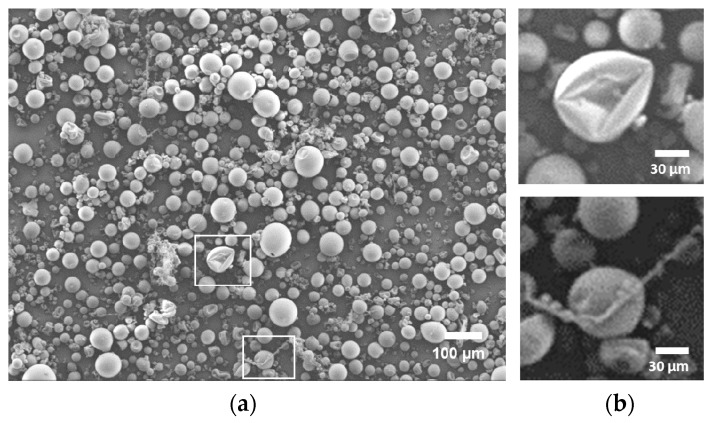
(**a**) Micrograph of spray-dried PSU from a 12 wt % solution in DMF; (**b**) insets show a magnification of a collapsed structure (above) and string-like structures (below).

Upon inspection with SEM microscopy (see Figure 2) it is clear that the lion′s share of the particles produced by spray drying consist of microspheres. A small portion of the particles exhibit either a collapsed or a string-like structure (see Figure 2b), which can be caused by too-high solution viscosity, causing bad jetting behavior.

**Figure 3:**
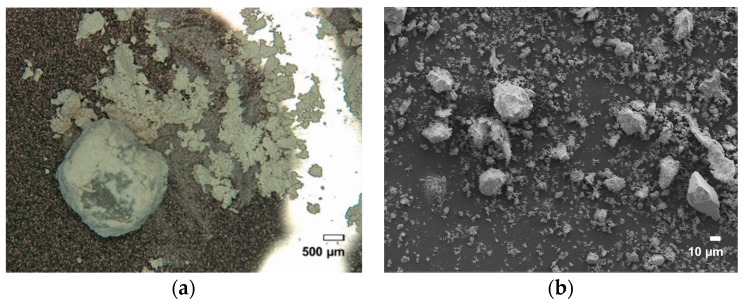
(**a**) Microscopic image of PSU taken after 10 min ball milling with (**b**) enlargement by SEM of the fractionated powder.

After 10 min of ball milling, the PSU pellets had already partly fractionated into a very fine powder (Figure 3a). SEM investigation of this fine powder revealed that it is neither spherical nor of the required size (Figure 3b). Instead, angular structures were apparent, which was to be expected with this technique. Further milling (sampling after every 10 min for 4 h) caused the remaining unfractionated material to pulverize with the same result. Additionally, the powder started to discolor with every fractionation step, suggesting degradation. It is widely known that ball milling is prone to induce degradation in polymeric material [11,27]. This will be further discussed in the section regarding the DSC and GPC results.

**Figure 4:**
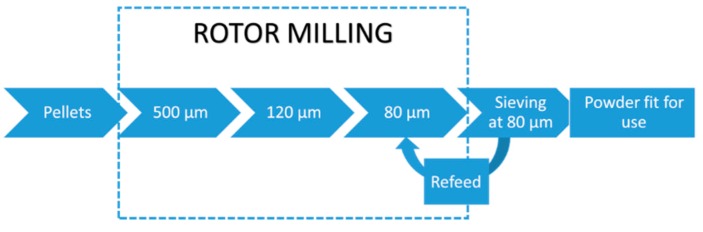
Schematics of the rotor milling process with the three-step refinement. After milling, the powders are sieved and the redundant powder is re-fed to the rotor miller for further refinement.

**Figure 5:**
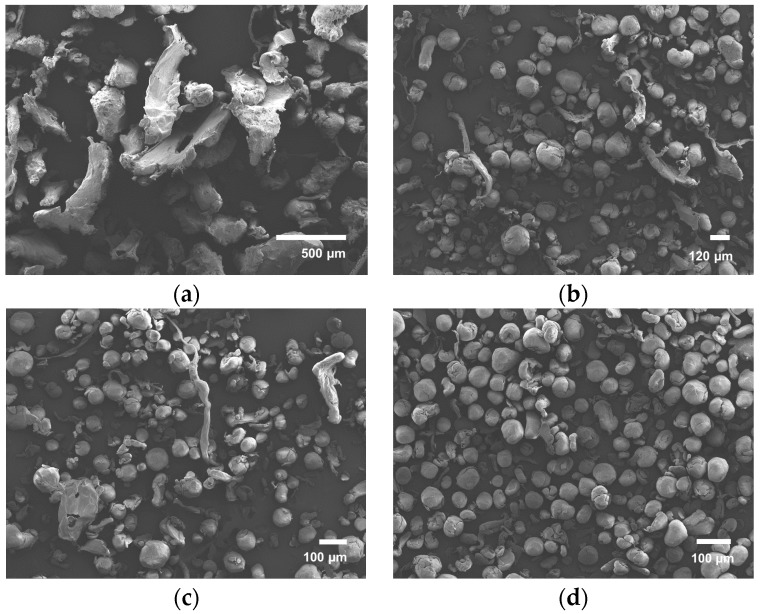
(**a**) Coarse powder by pulverization of pellets at 500 μm; (**b**) First refinement step by pulverisation of coarse powder at 120 μm; (**c**) Second refinement step by further pulverization of the refined powder until 80 μm and (**d**) Final powder after additional sieving at 80 μm.

Via a three-step communition process, the pellets are rotor milled to powder form (see Figure 4). First, the pellets are pulverized in the rotor mill using a sieve of mesh size 500 μm to form a coarse powder of very irregular structures with often little to no sphericity (Figure 5a). Structures were found to have dimensions of around 500 μm with some spherical particles of large size (approximately 150 μm) and large thread-like structures. This step was performed primarily to prevent the pellets from plasticizing against the high speed rotating mill. This powder was then further subjected to a refinement step in which the powders were further pulverized to 120 (Figure 5b) and 80 μm (Figure 5c) using sieves of the same mesh size. The final powder was sieved again at 80 μm to clear out the remaining non-spherical structures (Figure 5d). These were then re-fed to the Pulverisette. Subjecting this powder to a refinement step by which the powder was sequentially added to the Fritsch Pulverisette 14 with decreasing sieve sizes resulted in surprising results. A definite improvement in particle morphology was observed. Moreover, particle size progressively approached the aspired range of 45–90 μm with each refinement step. Particle size distributions are noted in Section 3.4.

### 3.4. Particle Size Distribution (PSD)

**Figure 6:**
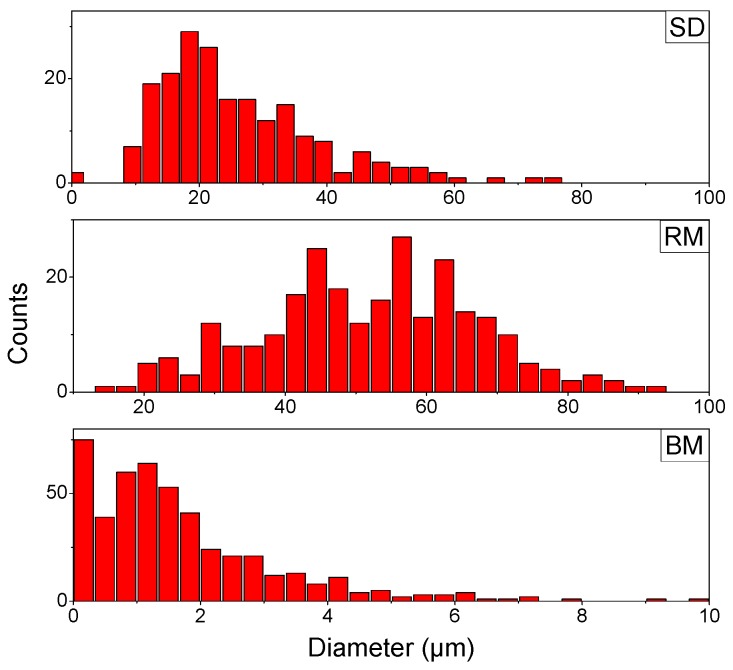
Particle Size Distribution (PSD) of the different processed samples. Spray-Dried (**SD**) sample at best parameter settings, Rotor Milled (**RM**) powder subjected to the three-step refinement process with final sieving step, and Ball Milled (**BM**) sample after 10 min (fractionated fine powder).

Micrographs of the produced powders with their corresponding PSD analysis using Image J are presented in Figure 6. Though spray dried particles are predominantly spherical, the mean particle size is only 26.1 μm with a standard deviation of 12.8 μm. The particle size curve is slightly skewed to the higher diameter range (see Figure 6, SD). Analysis of the fractionated powder obtained by ball milling reveals a mean particle size of 1.7 μm and standard deviation of 1.5 μm (see Figure 6, BM). The pellets have the tendency to fractionate rapidly into a very fine powder that tends to discolor after 20 min, and a large part of larger not-fully-fractionated pellets of millimeter size remain, making this process less appealing. When observing the rotor milled sample, no discoloration was noticed with each of the subsequent processing steps. The adjusted cooling procedure may play a role in this regard. PSD analysis performed on the powder of the first refinement step revealed a mean size of 115 μm with a standard deviation of 23 μm. Further refinement through milling reduced the size of the particles while preserving the spherical form. Particle size investigation of the final powder (see Figure 6, RM) revealed a mean size of 51.8 μm with a standard deviation of 15.2 μm, which is ideal for sintering experiments.

As the rotor milling process includes three milling rounds and spray drying two heat treatments of the polymer (dissolution by reflux and the spraying process), investigation of degradation is strongly advised. No discoloration was visible during processing with both methods, which lead us to believe that no degradation occurred during treatment of the polymer. DSC and GPC measurements were performed next to provide certainty on the matter (see Section 3.5 and Section 3.6).

### 3.5. GPC Measurements

Samples investigated concerned the ball milled sample which was left to mill for 10 min, the rotor milled sample going through the three-step communition process, the spray dried sample at best parameter settings and the unprocessed PSU as a reference sample.

**Figure 7:**
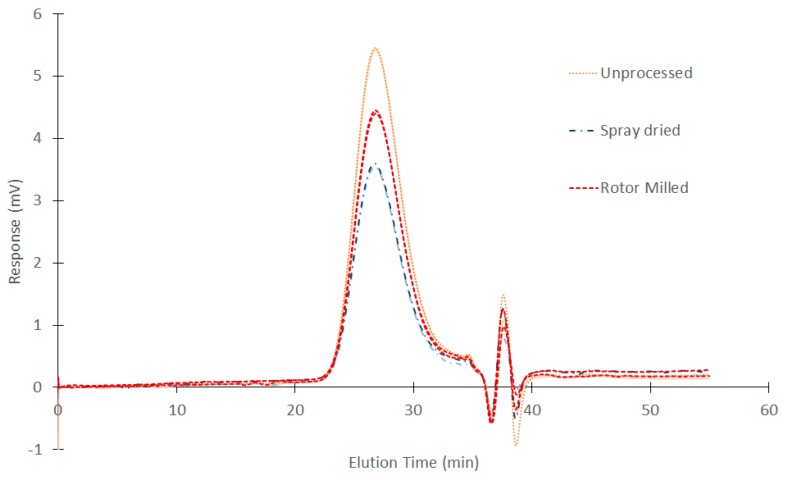
GPC measurements on unprocessed PSU, Rotor Milled powder subjected to the three step refinement process with additional sieving at 80 μm and spray-dried PSU obtained at the best parameter settings. The second measurements are represented in a lighter shade of the representative color.

The GPC curves are depicted in Figure 7 with respective molecular weights and polydispersity described in Table 2. No substantial shift in elution peaks is apparent, suggesting no significant degradation phenomena. When looking at the molecular weights calculated from these chromatograms, a slight increase in molecular weight is noticeable for the spray-dried sample. Although this is a very small change (approx. 2% and 3% for weight and number average molecular weight, respectively) this could suggest crosslinking due to degradation. As for the ball milled sample, dissolving the sample material for analysis through GPC seemed to be impossible, pointing towards severe crosslinking through degradation upon ball milling.

### 3.6. DSC Measurements

**Figure 8:**
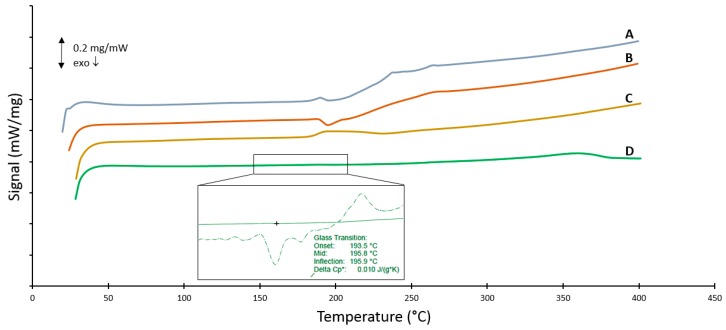
First heating run of DSC measurements on (**A**) spray-dried powder obtained at best parameter settings; (**B**) rotor milled powder subjected to the three step refinement process with additional sieving at 80 μm; (**C**) virgin PSU and (**D**) fine powder obtained after 10 min of ball milling the PSU pellets.

**Figure 9:**
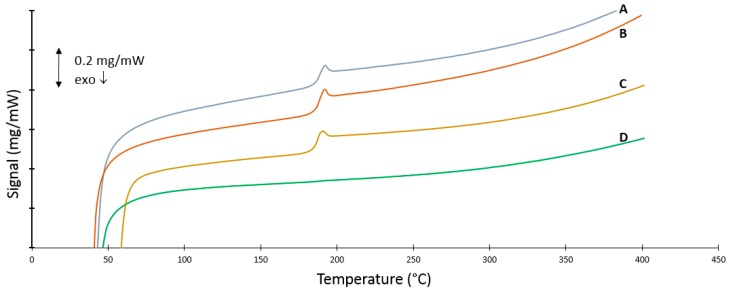
Second heating run of DSC measurements on (**A**) spray-dried powder obtained at best parameter settings, (**B**) rotor milled powder subjected to the three step refinement process with additional sieving at 80 μm, (**C**) virgin PSU and (**D**) fine powder obtained after 10 min of ball milling the PSU pellets.

As PSU is an amorphous polymer, we would expect the corresponding curves to be quite flat with the only change in baseline being the glass transition temperature of the polymer itself. The glass transition temperature represents the reversible transition in the amorphous regions within fully amorphous or semi-crystalline materials from a hard and relatively brittle “glassy” state into a molten or rubber-like state as a critical temperature is reached. The thermograms of the first and second heating run on all samples are depicted in Figure 8 and Figure 9, respectively. The DSC measurements show that the different processes have a different effect on the thermal behavior of PSU. The thermogram of virgin PSU (Figure 8C) exhibits a glass transition temperature at 189 °C followed by enthalpic relaxation, which is also expressed in the second heating run (Figure 9C). In the case of high-energy ball milling (Figure 8D), a broad endothermic peak is observed that exhibits a maximum at 359 °C. Furthermore, a glass transition temperature which is almost indistinguishable appears at 196 °C. The inset of Figure 8D depicts this by the change in the second derivative of the baseline. The significant shift in *T*_g_ is indicative of a certain degree of degradation occurring during the processing of PSU. The second heating run (Figure 9D) reveals that this broad endothermic peak is lost in the second heating run and might suggest an ordered state due to the processing method. This hypothesis is further tested by XRD measurements on the treated samples in Section 3.7. The second heating run of the ball milled sample also reveals a sternly diminished glass transition, confirming severe degradation. A different trend is found when looking at the rotor milled PSU (Figure 8B). A clear *T*_g_ at approximately 189 °C is visible, followed by a slight endothermic peak going from 210 °C to 236 °C. The peak, as was the case with ball milling, disappears upon heating above the endotherm and reappears in the second heating run, suggesting orientation imposed by the processing method onto the polymer chains. However, the *T*_g_ remains visible and stable upon the second run, indicating no severe degradation occurring in the process. With spray drying, the PSU was first dissolved, then atomized and dried to form spherical particles. Samples were consequently dried for two days in a vacuum oven at 50 °C in order to remove any residual solvent. A clear *T*_g_ is visible at 188 °C with two small endothermic peaks at 237 and 262 °C in the first heating run (Figure 8A). These peaks disappear upon heating above their offset temperature and also suggest a form of orientation imposed on to the polymer chains by the processing method. The second heating curve (Figure 9A) shows a clear *T*_g_ again at 189 °C, in accordance with the unprocessed PSU.

### 3.7. XRD Measurements

**Figure 10:**
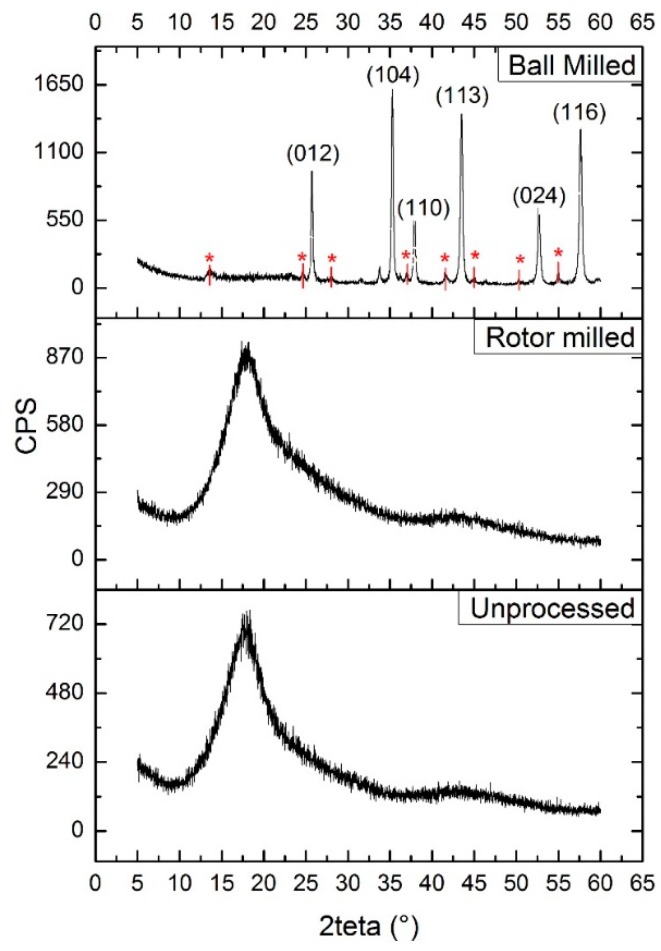
XRD diffractograms of the fine powder obtained after 10 min of ball milling, rotor milled powder subjected to the three step refinement process with additional sieving at 80 μm, and unprocessed PSU pellets. Small peaks marked with an asterisk are believed to be attributed to the orientation induced in PSU by the milling method.

X-ray diffraction measurements were performed to test the hypothesis that the chosen mechanical processing method could impart some orientation into the molecular structure of the material. As PSU is amorphous in its unprocessed forms, we would expect the diffractograms of the treated samples not to show any distinct peaks in the range measured. When looking at the diffractograms represented in Figure 10, the typical broad bands for amorphous polymers without any crystallinity are found. For both rotor milled and unprocessed PSU, this broad band shows a maxima at 1.5°. However, when looking at the ball milled sample, this broad amorphous band has disappeared and instead sharp peaks can be seen at 25.7; 35.3; 37.9; 43.5; 52.7; and 57.6 degrees. These diffractions are representative of the lattice planes (012); (104); (110); (113); (024) and (116) of alumina (of which the ceramic cups of the ball mill are composed [28]), indicative of contamination during milling. However, next to the indexed peaks, smaller diffractions are visible (marked with an asterisk in Figure 10), which are indicative of an ordered structure that is able to reflect the incident X-rays. Finally, the broad amorphous peak which should be present around 17.5° is strongly diminished, again confirming severe degradation. The spray-dried and rotor milled samples did not show any change in their diffractogram from that of the unprocessed PSU; for this reason, only the diffractogram of the rotor milled sample is shown.

## 4. Discussion

### 4.1. Morphology

When comparing the morphology of the powders obtained by the different processing methods, ball milling does not meet the dimensional requirements that one looks for in SLS powders. Particles are neither spherical nor in the right size range with a mean diameter of 1.7 μm and standard deviation of 1.5 μm. Throughout the milling process, particles fractionated in angular structures of variable sizes, often too small. Fractionation in submicron particles presents itself as a problem for processing PSU. Furthermore, a discoloration was noticed with each sampling step taken, indicating degradation, which is confirmed by the DSC, XRD, and GPC measurements discussed further on.

Although ball milling is believed to have a negative influence on the molecular weight of the polymer to pulverize, it was thought that a novel kind of mechanical milling with shorter contact time might prove to be a faster and cleaner way to achieve the desired goals. Rotor milling yields better results regarding the morphology of the particles, giving microspheres of 51.8 μm with a standard deviation of 15.2 μm. In order to explain the spherical morphology, one has to look at the rotor milling process: in rotor milling, breakage occurs by a rotating 12-ribbed cutting blade that impacts against the particles at high speeds. Upon impact, the kinetic energy of the impact tool gives rise to deformation in the contact area. As with ball milling, breakage will occur when the elastic stress of the particle reaches a critical level. Fractionation is mediated by the occurrence of incipient cracks as a result of high local stresses. Contrary to ball milling, an additional shearing force is present in rotor milling between the rotating blade and sieve ring. Particles endure an extra deformation by the rotating motion of the rotating blades causing the particles to round off.

**Figure 11:**
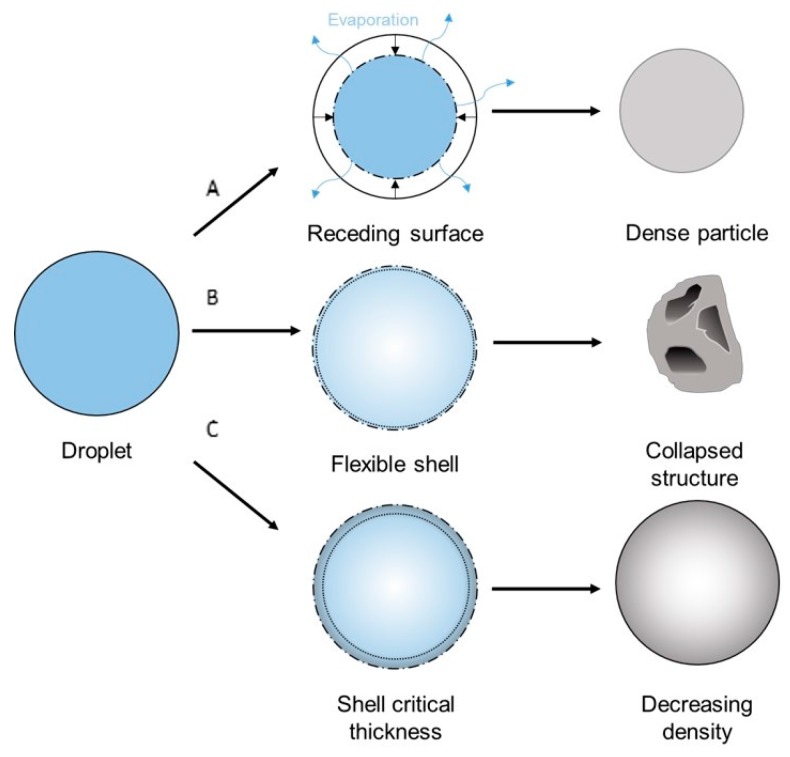
Evaporation model that illustrates possible morphology occurrence depending on parameters used.

Spray-drying the polymer solution also resulted in microspheres, albeit at smaller particle size: 26.1 μm mean diameter with a standard deviation of 12.8 μm. Further increase of the particle size is possible by increasing nozzle size and drying chamber, which implies upscaling from the Buchi B290 to a larger model, as larger droplet size is required to obtain larger particles [29]. This is not possible with the current setup, yet an ample amount of pilot scale models exist that could meet this demand. Though the main part of the particles consists of microspheres, inconsistencies in the form of collapsed and string like structures were still present on a smaller scale. These inconsistencies can be ascribed to bad jetting behavior caused by too-high solution viscosity in the latter case. The former case can be explained by the low diffusion rate of the polymers. Polymer solutions generally exhibit a large Peclet number, which is related to a low diffusion rate. A low solute diffusion rate can result in skim or shell formation, depending on the parameters used. Vehring *et al.* [30] described these effects at length through both a numerical and an analytical model. The evaporation model is illustrated in Figure 11. Low molecular weight solutions benefit from a large diffusion rate that allows the solutes to migrate relatively easily inside the droplet. When a droplet is drying, the solvent evaporates at the surface of the droplet, creating a concentration gradient that diminishes to the inside of said droplet. As more solvent evaporates, the surface of the droplet recedes and a saturation occurs, leading to homogeneous precipitation throughout the droplet. This should lead to a dense particle (A in Figure 11). However, when dealing with large molecular weight particles, the diffusion rate is slow compared to the speed of the receding surface, causing a thin shell to be formed. A number of situations may occur; if the evaporation rate is high, a thin shell is formed that is not mechanically stable enough to sustain itself (B in Figure 11). As the solvent evaporates further, the shell will collapse, forming toroid-like or wrinkled structures. On the other hand, if the evaporation rate is high, the shell will attain a critical thickness stable enough to sustain itself (C in Figure 11). In this case, the evaporation continues by migration of the solvent molecules through capillary flow, and precipitation occurs, resulting in a density of the particle that slightly decreases inwards.

### 4.2. Physicochemical Properties

**Figure 12:**
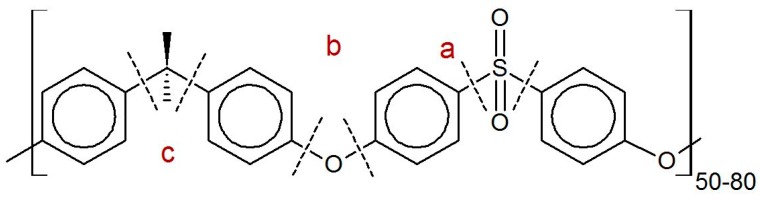
Possible degradation mechanisms of polysulfone given: (**a**) crosslinking by phenylation of phenyl radical formed by cleavage of C–S bond; (**b**) intramolecular phenylation by hydrogen abstraction by the phenyl radical; (**c**) β-scission of isopropylidene radical following H-abstraction [31,33,35].

GPC measurements of both spray-dried and rotor milled samples revealed no substantial shift in elution peaks, leading us to believe that no significant degradation had occurred as a result of the processing method. The slight change in *M*_n_ and *M*_w_ for the spray drying method could indicate that spray drying is more sensitive to degradation, as it shows the largest change in molecular weight compared to the unprocessed polymer. This was to be expected, as the PSU undergoes two thermal treatments in order to be processed to powder form. Firstly by dissolving by reflux and secondly by the spray-drying process. The degradation process of polysulfone materials has been extensively studied since their discovery. Polysulfones tend to degrade by heteroatom bridge cleavage, causing the material to further degrade by random chain scission, crosslinking, and possibly the β-scission of the isopropylidene moiety [31,32,33,34,35]. Possible degradation mechanisms of polysulfone are given in Figure 12. In the case of the ball milled sample, this premise is justified by the fact that the polymer was unable to dissolve for GPC measurement, by the discoloration seen while ball milling, and by DSC and XRD measurements discussed in Section 3.6 and Section 3.7.

The DSC measurements reveal the influence that the processing methods impart on the thermal properties of the polymer. Both rotor milling and spray-drying show no significant change in *T*_g_; therefore, no severe degradation is assumed. However, some orientation is visible in both powders by the appearance of small endothermic peaks. This could be ascribed to the processing methods; in the case of rotor milling, the polymer chains of the material stretch out when the pellets of powder impact on the rotor blades and get further sheared apart between the blades and the sieving ring. Upon the first heating run, an endothermic peak is then visible, ascribed to the relaxation of the chains. This orientation in the material can also explain the endothermic peaks in the thermogram of the spray-dried sample, as the polymers experience high shearing rates while jetting into the drying chamber.

The significant change in glass transition temperature for high energy ball milling is indicative of severe degradation. Mechanical milling could conceivably cause chain scission in the polymer molecules with the creation of free radicals, which may then react with other polymer chains, causing crosslinking. Though chain scission should cause a decrease in *T*_g_, the grafting or crosslinking process should increase its value. Together with the fact that the milled powder was unable to be dissolved for GPC measurements and the discoloration noticed during each sampling step, degradation by crosslinking seems a logical explanation. The appearance of the endothermic peak in the thermogram of the ball milled sample has not yet been fully explained. Bai *et al*. describe a biaxial stretching and orientation of the polymer chains that occurs due to the compressive and shearing deformation of the collisions with the balls and crucible of the ball mill [7]. This “ordered amorphous” state could be an explanation for the high temperature endotherm. The disappearance of the endothermic peak in the second heating run further confirms this hypothesis of the structured amorphous state which becomes fully amorphous again with adequate thermal energy.

XRD measurements reveal no change in diffractogram for the processed samples of PSU by rotor milling or spray drying; from the lack of change in the broad amorphous band and the absence of any other significant peaks that could imply an ordered structure, one can conclude that the processing method did not impart any structural changes in the polymer chains. Looking at the high energy ball milled structure, however, the broad amorphous peak that should be present around 17.5° is strongly diminished, confirming severe degradation. This possibly confirms the hypothesis of an ordered amorphous structure imparted by the processing method.

## 5. Conclusions

Polysulfone pellets were processed into powder form for the use in Selective Laser Sintering. Of the processing methods tested, rotor milling and spray-drying displayed the best results. Rotor milling could successfully be used in a three step refinement process to produce particles of desired size (51.8 ± 15.2 μm) and morphology. In the case of spray-drying, particles were prominently spherical, yet particle size was deemed a bit lacking (26.1 ± 12.8 μm). Further increase of particle diameter was hindered by the dimensions of the laboratory spray dryer, and further optimization in this regard is necessary. This problem can conceivably be solved by the use of a pilot scale spray dryer with a larger drying chamber and bigger nozzle diameter. Ball milling proved not to be a suitable method of processing the PSU, as severe degradation occurred; moreover, particles were angular in shape and well below the target size range (1.7 ± 1.5 μm). GPC measurements revealed no significant degradation in both spray-drying and rotor milling processes. Both processing methods imparted some orientation to the polymer chains, causing extensive enthalpic relaxation visible in the DSC measurements. In the case of ball milling, a clear shift in glass transition temperature and an endothermic peak in the high temperature region were observed. XRD measurements related this to an ordered amorphous structure imparted on the material by the processing technique.

## Figures and Tables

**Table 1:** Shortlist of Hansen Solubility Parameter (HSP) parameters of polysulfone (PSU) and possible compatible solvents [24].

Solvents	δt (MPa^1/2^)	δd (MPa^1/2^)	δp (MPa^1/2^)	δh (MPa^1/2^)
**Polysulfone**	23.6	19.8	11.2	6.2
**Chloroform**	18.9	17.8	3.1	5.7
***N*,*N*-Dimethylformamide**	24.9	17.4	13.7	11.3
***N*,*N*-Dimethylacetamide**	22.8	16.8	11.5	10.2
**Tetrahydrofuran**	19.5	16.8	5.7	8.0

**Table 2:** Gel Permeation Chromatography (GPC) measurements of virgin and processed samples.

Processing method	*M*_w_^1^	*M*_n_^2^	Polydispersity
**Virgin/unprocessed**	60,318 ± 283	29,804.5 ± 146	2.02 ± 4
**Rotor milling**	60,293 ± 217	30,297.5 ± 1,272	1.99 ± 0.11
**Spray drying**	61,662 ± 110	30,803.5 ± 1,769	2.01 ± 0.09
**Ball milling**	n.a.^.3^	n.a.^.3^	n.a.^.3^

^1^ Weight average molecular weight; weighted molecular weight according to weight fractions; ^2^ Number average molecular weight; total weight of the sample divided by the number of molecules in sample; ^3^ n.a. = not applicable.

## Abbreviations

The following abbreviations are used in this manuscript:

AM	Additive Manufacturing
DIPS	Diffusion Induced Phase Separation
DMF	*N*,*N*-Dimethylformamide
EPS	Evaporation Induced Phase Separation
HDT	Heat Deflection Temperature
HSP	Hansen Solubility Parameter
PSD	Particle Size Distribution
SD	Spray Drying
SLS	Selective Laser Sintering
TIPS	Thermal Induced Phase Separation
